# Addisonian Crisis after Missed Diagnosis of Posttraumatic Hypopituitarism

**DOI:** 10.3390/jcm4050965

**Published:** 2015-05-15

**Authors:** Christine Streetz-van der Werf, Wolfram Karges, Marcus Blaum, Ilonka Kreitschmann-Andermahr

**Affiliations:** 1 Division of Endocrinology and Diabetes, RWTH Aachen University, 52062 Aachen, Germany; E-Mails: cstreetz-vanderwerf@ukaachen.de (C.S.W.); wkarges@ukaachen.de (W.K.); 2 Department of Neuroradiology, RWTH Aachen University, 52062 Aachen, Germany; E-Mail: mblaum@ukaachen.de; 3 Department of Neurosurgery, University Hospital Essen, 45147 Essen, Germany

**Keywords:** traumatic brain injury, hypopituitarism, neuroendocrine dysfunction, hypocortisolism, Addisonian crisis

## Abstract

We report a case of a previously undiagnosed panhypopituitarism initially presenting as a full-blown Addisonian crisis with hypoglycemia, hyponatremia, hypotension and neuropsychological symptoms, more than 30 years after a severe traumatic brain injury (TBI). The patient also displayed clearly visible pathognomonic clinical signs of long-standing pituitary dysfunction. The case highlights the importance of being aware of endocrine sequelae even decades after serious TBI.

## 1. Introduction

In the last two decades, traumatic brain injury (TBI) as a major and perhaps formerly underestimated cause of hypothalamic-pituitary dysfunction has been intensely researched and discussed in the medical literature (for an overview see [1,2,3]). Despite the attention this topic receives in scientific journals, routine testing for endocrine dysfunction in TBI survivors is still rarely performed. Moreover, patients who have sustained TBI often present with complaints and symptoms such as depression, mental slowing and reduced physical performance that may be ascribed to the sustained brain damage itself, potentially masking the endocrine sequelae of the trauma. It is therefore likely that many cases of posttraumatic hypopituitarism remain undiagnosed and untreated, with potentially life-threatening consequences for the affected patients as illustrated in the reported case.

## 2. Case Report

Following dental surgery scheduled as a routine outpatient procedure, a 49 year-old male patient developed nausea, vomiting, and general fatigue, leading to admission to a community hospital for suspected acute gastroenteritis. He further developed mental confusion, arterial hypotension (89/46 mmHg), hyponatremia (plasma sodium, 117 mmol/L, normal 132–148 mmol/L) and hypoglycaemia (fasting plasma glucose 3.2 mmol/L, normal 3.3–5.3 mmol/L), and had to be transferred to the intensive care unit.

**Figure 1:**
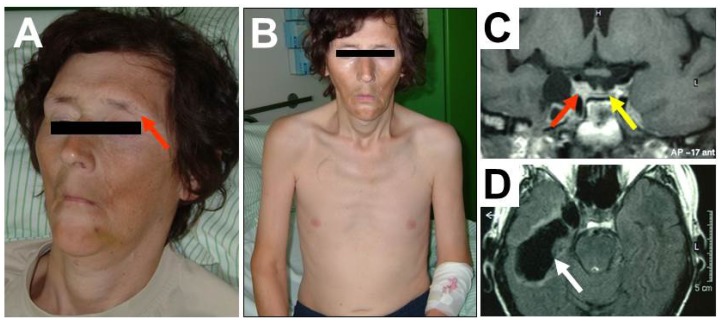
Forty-nine year-old patient with severe posttraumatic hypopituitarism. Clinical appearance, showing lack of facial hair, dehydration, Queen Anne’s sign (panel **A**), pale skin, muscular atrophy, loss of body hair and anorexia (panel **B**). T1-weighted coronal MRI of the pituitary region, with flattened pituitary gland at the bottom of the sella (panel **C**, yellow arrow) and contusional defects in the temporal lobe (panel **D**, white arrow). Tissue adjacent to the right cavernous sinus (panel **C**, red arrow) compatible with dislocated pituitary tissue after fracture of the middle cranial fossa.

Physical examination revealed dehydration, severe hypogonadism with loss of pubic, facial and axillary hair (Figure 1A), Queen Anne’s sign (loss of the outer third of the eyebrows as a clinical sign of hypothyroidism [4] (see red arrow Figure 1A), pale and waxen skin, muscular atrophy (Figure 1B) and low testicular volume. The patient’s initial basal hormone levels are presented in Table 1.

After fluid, electrolyte and hydrocortison replacement, the condition of the patient improved.

Subsequent dynamic endocrinological testing with insulin tolerance testing (ITT) revealed insufficient rise of growth hormone (maximum, 0.4 µg/L), cortisol (maximum, 49 nmol/L), and ACTH response (maximum, 10.3 pmol/L), confirming severe hypopituitarism. There was no clinical evidence of diabetes insipidus. Osteodensitometry (DEXA) of the lumbar spine showed a T-score of −3.72, indicating secondary osteoporosis.

**Table 1:** Results of the basal hormone levels of the patient.

	Laboratory Values	
Variables (Units)	Results	Normal Range
Serum free thyroxine, fT4 (pmol/L)	2.6	10.3–23.2
Thyroid stimulating hormone, TSH (mU/L)	3.56	0.27–4.2
Total testosterone (nmol/L)	<0.1	9.9–27.8
Sex hormone binding globulin, SHGB (nmol/L)	94.5	15–48
Luteinizing hormone, LH (mU/L)	<0.1	1.7–8.6
Follicle stimulating hormone, FSH (mU/L)	0.5	1.5–12.4
Prolactin (mU/L)	19	86–324
Insulin-like growth factor-1, IGF-1 (nmol/L)	<3.3	12.2–32.8
Serum cortisol * (nmol/L)	47	171–536
Adrenocorticotroph hormone, ACTH (pmol/L)	1.7	1.5–14.7

* Serum cortisol was measured at 8:00 a.m.

Thirty-one years earlier the patient had suffered severe traumatic brain injury (TBI) from a car accident, with a fracture of the skull base and prolonged coma. In the years after the injury beard growth decreased gradually and the patient had reported to have unsuccessfully sought urologic advice for impaired libido and sexual dysfunction. Even shortly before the Addisonian crisis, the patient reported to have been able to work full-time in an office job and do easy kinds of sports (power yoga). He had a female life-companion, but no biological children and was taking no regular medication. Clinical history revealed no other anamnestic information beside the TBI (no operations, severe illnesses, or further head and neck injuries) that could be related to a loss of pituitary function. MRI of the head and pituitary was performed at the time of clinical deterioration, showing a flattened pituitary (Figure 1C) and temporal contusional defects (Figure 1D).

In the months after continuous replacement with thyroid hormones, hydrocortisone and recombinant growth hormone, the condition of the patient improved markedly. However, exposure to transdermal testosterone replacement in a very low dose caused a psychotic episode, necessitating inpatient psychiatric treatment. The symptomatology resolved after discontinuation of testosterone, but the patient refused re-exposure ever since. Today, he is able to lead an independent life with a normal physical and psychological capacity, works full-time and has a stable partnership. Next to hormone replacement therapy, he is currently on combined calcium/vitamin D3 supplementation. Due to nocturnal epilepsy, diagnosed in 2011, most likely a late sequel of the TBI, he is on anticonvulsant medication (levetiracetam).

## 3. Discussion

The first description of severe posttraumatic hypopituitarism dates back almost a century [5] and relates the case of a 48-year old man who suffered a contusion of the brain with skull base fracture due to having his head squashed between two railway buffers. Similarly to the currently reported patient, he subsequently developed loss of secondary hair growth and progressive cachexia and was investigated for these problems many years after the injury. Despite this and other early clinical descriptions of posttraumatic hypopituitarism [6], TBI as a clinically relevant cause of hypothalamic-pituitary dysfunction has only recently come into the focus of attention ([1,3,6] and the current JCM issue). The reported prevalences of posttraumatic hypopituitarism vary widely, and depend on the subgroups studied and the methods employed to ascertain the diagnosis. Moreover, hormone deficiencies are most often partial with only one or two affected axes. Since individuals after TBI commonly suffer from a myriad of neurological, somatic and psychiatric sequelae of the trauma, it is difficult to ascertain which of the presented clinical symptoms may also be caused or aggravated by neuroendocrine dysfunction. A recent study from a Danish group found only a very limited relationship between hormonal dysfunction and quality of life or fatigue 2.5 years (median) after TBI [7]. These controversies and the lack of established, clear-cut risk markers for posttraumatic anterior hypopituitarism may account for the fact, that routine testing for endocrine dysfunction in TBI survivors is not widely performed in clinical practice [8]. Such a clinical attitude may, however, be deleterious for patients as the one described in our clinical vignette. The presented case also serves as reminders of the classic clinical features of long-standing neuroendocrine dysfunction and of the fact, that patients with adrenal crisis often present with hypotension and vomiting, leading to the misdiagnosis of gastrointestinal disease [9]. Hypopituitarism is not an infrequent sequel of TBI and it should not be forgotten, that it can have severe and life-threatening consequences, as shown here, if failed to diagnose.

## 4. Conclusions

Adrenal crisis is a feared and potentially lethal complication of primary but also secondary hypoadrenalism. Clinicians must be aware that TBI is a potential cause of such severe hypopituitarism.

## References

[1] Fernandez-Rodriguez E., Bernabeu I., Castro A.I., Casanueva F.F. (2015). Hypopituitarism after traumatic brain injury. Endocrinol. Metab. Clin. North Am..

[2] Schneider H.J., Aimaretti G., Kreitschmann-Andermahr I., Stalla G.K., Ghigo E. (2007). Hypopituitarism. Lancet.

[3] Schneider H.J., Kreitschmann-Andermahr I., Ghigo E., Stalla G.K., Agha A. (2007). Hypothalamopituitary dysfunction following traumatic brain injury and aneurysmal subarachnoid hemorrhage: A systematic review. JAMA.

[4] Lane Furdell E. (2007). Eponymous, anonymous: Queen Anne’s sign and the misnaming of a symptom. J. Med. Biogr..

[5] Cyran E. (1918). Pituitary damage due to basal skull fracture (in German). Dtsch. Med. Wochenschr..

[6] Witter H., Tascher R. (1957). Hypophyseal-hypothalamic symptoms after blunt cranial trauma (in German). Fortschr. Neurol. Psychiatr..

[7] Klose M., Stochholm K., Janukonyte J., Christensen L.L., Cohen A., Wagner A., Laurberg P., Christiansen J.S., Andersen M., Feldt-Rasmussen U. (2015). Patient reported out-come in posttraumatic pituitary deficiency: Results from the Danish National Study on posttraumatic hypopituitarism. Eur. J. Endocrinol..

[8] Agha A., Thompson C.J. (2006). Anterior pituitary dysfunction following traumatic brain injury (TBI). Clin. Endocrinol..

[9] Arlt W., Allolio B. (2003). Adrenal insufficiency. Lancet.

